# Application of Bio-Fertilizers Improves Forage Quantity and Quality of Sorghum (*Sorghum bicolor* L.) Intercropped with Soybean (*Glycine max* L.)

**DOI:** 10.3390/plants12162985

**Published:** 2023-08-18

**Authors:** Elnaz Sadafzadeh, Abdollah Javanmard, Mostafa Amani Machiani, Adriano Sofo

**Affiliations:** 1 Department of Plant Production and Genetics, Faculty of Agriculture, University of Maragheh, Maragheh P.O. Box 55136-553, Iranamani0056@gmail.com (M.A.M.); 2 Department of European and Mediterranean Cultures: Architecture, Environment, Cultural Heritage (DiCEM), Università degli Studi della Basilicata, Via Lanera 20, 75100 Matera, Italy

**Keywords:** crude protein, forage quality, intercropping, sustainable agriculture, total digestible nutrients

## Abstract

In recent years, application of bio-fertilizers (BFs) in intercropping systems has become known as one of the main sustainable and eco-friendly strategies for improving the quantity and quality of forage crops. In order to evaluate the forage quantity and quality of sorghum intercropped with soybean, a two-year field experiment was carried out as factorial based on a randomized complete blocks design (RCBD) with three replications. The first factor was different cropping patterns including soybean monocultures with densities of 40 and 50 plants m^−2^ (G40 and G50), sorghum monocultures with densities of 10 and 15 plants m^−2^ (S10 and S15) and intercropping of two plants with the mentioned densities. The second factor was non-application (control) and application of bio-fertilizers. The results demonstrated that the highest dry forage yield of sorghum (21.22 t ha^−1^) was obtained in monoculture conditions with density of 15 plants m^−2^ and inoculation with bio-fertilizer (S15+BF). The maximum crude protein (CP = 149.6 g kg^−1^ DM), ash (113.2 g kg^−1^ DM), water soluble carbohydrates (WSC = 251.16 g kg^−1^ DM), dry matter intake (DMI = 26.83 g kg^−1^ of body weight), digestible dry matter (DDM = 668.01 g kg^−1^ DM), total digestible nutrients (TDN = 680.42 g kg^−1^ DM), relative feed value (RFV = 142.98%) and net energy for lactation (NEL = 1.625 Mcal kg^−1^) were observed in the intercropping of S10G50 inoculated with BF. Interestingly, application of bio-fertilizers enhanced the content of CP, ash, WSC, DMI, DDM, TDN, RFV and NEL by 7.5, 8, 11.7, 3.6, 2.3, 12.3, 5.9 and 3.5% when compared with the control (non-application of bio-fertilizers). In all intercropping patterns, the total land equivalent ratio (LER) value was greater than one, representing the advantage of these cropping patterns in comparison with sorghum monoculture. The highest total LER was recorded in the intercropping of S15G40 and S10G50 following application of BF. Additionally, the highest monetary advantage index (MAI) was calculated in the intercropping of S15G40+BF. Generally, it can be concluded that the intercropping of S10G50 along with bio-fertilizer inoculation could be suggested as an eco-friendly strategy for improving the forage quantity and quality under low-input conditions.

## 1. Introduction

Conventional agricultural systems are designed based on two goals: increasing crop productivity and higher income. The success rate of these agricultural systems depends on the excessive use of chemical inputs and newly bred varieties. The excessive application of chemical inputs such as synthetic fertilizers, pesticides, etc., can cause many negative problems, including environmental contamination and harmful effects on human health and other living organisms in the ecosystem [1,2]. In addition, the development of these systems decreases biodiversity and plant’s productivity in the long term [2].

To tackle these problems, it seems necessary to use sustainable systems to reduce the negative effects of chemical inputs and also improve the quantity and quality of agricultural products [3]. Intercropping, as an eco-friendly and cleaner production system, is a type of multiple-cropping system in which two or more crops are cultivated in the same parcel of land at the same time [4,5]. This cropping pattern is a very well-known agricultural system in developing countries due to some of its comparative advantages such as enhancing environmental resources (water, nutrients, solar radiation, etc.) use efficiency, increasing competitive potency for weed control, reduction in pathogens and pests and improvement the quantity and quality of the crops [6,7,8]. 

Sorghum (*Sorghum bicolor* L.) is an annual plant belonging to the cereal family. Sorghum, known as a dual-purpose crop (seed and fodder), after wheat, barley, rice and corn is the fifth most important grain in the world and plays an important role in animal nutrition [9]. Sorghum can be used as a suitable forage crop in arid and semi-arid regions due to its lower transpiration rate and consumption of 25% less water than forage maize [10]. In recent years, the cultivation area of forage sorghum in Iran has increased to more than 25 thousand hectares. Although sorghum has a high ability to produce dry matter, the fodder from this plant is poor in protein. Protein is necessary for the growth and production of sufficient milk by livestock and the activity of bacteria in the digestive system of ruminant animals, which are responsible for digesting the fodder consumed by livestock [11]. Therefore, intercropping of sorghum with nitrogen-fixing plants is known as an eco-friendly strategy for improving the quantity and quality of this plant under sustainable agricultural conditions.

Forage soybean is known as one of the high-yielding and high-quality summer fodder plants and can be a good source of protein for feeding ruminants. As a forage crop, it has several advantages including high protein content at the seed filling stage, a wide range of growth stages suitable for harvest, efficient cover to reduce soil erosion and wide adaptability to different climatic zones. The cultivation of legume crops such as soybean can improve soil fertility by increasing soil nitrogen content through a biological nitrogen fixation process [12]. Previous studies have shown that the intercropping of nitrogen-fixing plants with non-legume forage plants can increase the growth and improve the quantity and quality of forage crops. Crusiol et al. [13] concluded that the protein concentration of Palisades grass (*Brachiaria brizantha*) leaves intercropped with soybean was 107–139 g kg^−1^ of dry matter, which is more than the minimum required protein (70 g kg^−1^ of dry matter) for maintaining the population of microorganisms in the animal’s rumen. Also, these researchers reported that despite the low soil nitrogen in this experiment, the dry matter and protein content of palisade grass increased in intercropping patterns. 

Application of bio-fertilizers is known as a sustainable solution for reducing and eliminating the chemical inputs in sustainable agricultural systems [14,15]. A large number of soil bacterial species that live in the rhizosphere of plants are able to improve plant growth by different mechanisms. These bacteria stimulate plant growth by producing various compounds, facilitating the absorption of elements, stabilizing atmospheric nitrogen, solubilizing minerals such as phosphate, and producing plant hormones such as auxins and gibberellins, which increase plant growth and productivity [16].

Forage shortage and low quality of available forages are known as main problems of livestock production in most parts of the world, including Iran. Previous results have shown that the usage of legume plants intercropped with non-legume forages enhanced the quantity and quality of forage [17,18,19]. In addition, the nutrient deficiency, especially in low-input conditions, and the negative impacts of excessive chemical fertilizers consumption with the aim of increasing plant’s productivity could endanger the health of livestock and decrease the quantity and quality of forage crops. Therefore, the experiment was aimed to investigate the effect of different intercropping patterns of soybean/sorghum along with bio-fertilizer application on the quantity and quality of forage sorghum.

## 2. Results

### 2.1. Sorghum Forage Productivity

**Figure 1:**
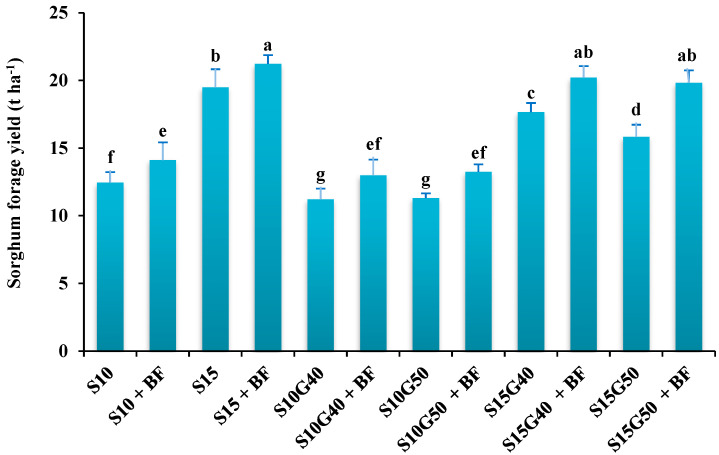
Sorghum forage productivity in different cropping patterns and bio-fertilizer application. Different letters indicate significant differences at the 5% level according to LSD test.

The highest dry forage yield of sorghum (21.22 t ha^−1^) was obtained in monoculture conditions with a density of 15 plants m^−2^ with bio-fertilizer application (S15+BF), which had no significant difference between S15G40+BF and S15G50+BF. The lowest dry forage yield of sorghum was achieved in the S10G40 and S10G50 intercropping without inoculation. The average dry forage yield of sorghum in intercropping patterns was reduced by 8.2 and 9.7% in comparison with S10 and S15 monoculture, respectively. Interestingly, the dry forage yield of sorghum was enhanced by 15.6% after inoculation with BF (Figure 1).

### 2.2. Soybean Forage Productivity

**Figure 2:**
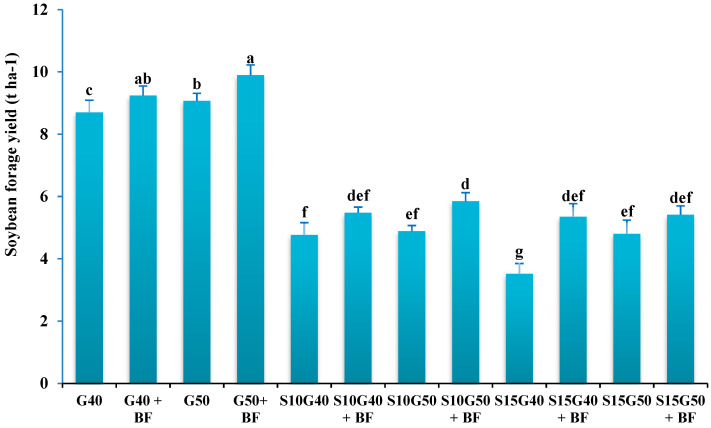
Soybean forage productivity in different cropping patterns and bio-fertilizer application. Different letters indicate significant differences at the 5% level according to LSD test.

The highest dry forage yield of soybean (9.9 t ha^−1^) was obtained in monoculture conditions with density of 50 plants m^−2^ with bio-fertilizer application (G50+BF). The lowest dry forage yield of soybean (3.52 t ha^−1^) was achieved in the S15G40 intercropping without inoculation. The average dry forage yield of soybean in intercropping patterns was reduced by 46.7 and 44.8% in comparison with G40 and G50 monoculture, respectively. Additionally, the dry forage yield of soybean was enhanced by 15.3% after inoculation with BF (Figure 2).

### 2.3. Crude Protein Content (CP)

The maximum CP content (149.6 g kg^−1^ DM) was measured in S10G50 intercropping inoculated with BF. In contrast, the minimum CP content of forage was observed in the sorghum monoculture without BF inoculation (S10 and S15). The average CP content in different intercropping patterns was enhanced by 43.3% in comparison with sorghum monoculture. Additionally, the CP content of forage was enhanced by 7.5% after inoculation with BF (Table 1).

### 2.4. Forage Ash

Among different treatments, the intercropping pattern of S10G50+BF produced the highest ash content of forage (113.2 g kg^−1^ DM), which had no significant difference with S10G50. The average ash content in different intercropping patterns was enhanced by 83.2% in comparison with sorghum monoculture. Also, the ash content of forage was enhanced by 8% after inoculation with BF (Table 1).

### 2.5. Acid Detergent Fiber (ADF)

The maximum ADF concentration of forage (377.43 g kg^−1^ DM) was achieved in the sorghum monoculture without BF inoculation (S15), which had no significant difference with S10. In contrast, the minimum ADF concentration (258 g kg^−1^ DM) was observed in the intercropping of S10G50+BF inoculation. The average ADF concentration in different intercropping patterns was reduced by 17.8% in comparison with sorghum monoculture. Additionally, the ADF concentration of forage decreased by 6.2% after inoculation with BF (Table 1).

### 2.6. Neutral Detergent Fiber (NDF)

Similar to ADF results, the maximum NDF concentration of forage (622.56 g kg^−1^ DM) was achieved in the sorghum monoculture without BF inoculation (S15). In contrast, the minimum ADF concentration (449.36 g kg^−1^ DM) was observed in the intercropping of S10G50+BF inoculation. The average NDF concentration in different intercropping patterns was reduced by 18.5% in comparison with sorghum monoculture. Additionally, the NDF concentration of forage decreased by 3.8% after inoculation with BF (Table 1).

### 2.7. Water Soluble Carbohydrate (WSC)

The maximum WSC content (251.16 g kg^−1^ DM) was measured in the intercropping of S10G50+BF, which had no significant difference with S10G40+BF. In contrast, the minimum WSC content was observed in the sorghum monoculture without BF inoculation (S15 and S10). The average WSC content in different intercropping patterns was enhanced by 18.4% in comparison with sorghum monoculture. Additionally, the WSC content of forage was enhanced by 11.7% after inoculation with BF (Table 1).

### 2.8. Dry Matter Intake (DMI)

The highest (26.83 g kg^−1^ of body weight) and lowest (19.30 g kg^−1^ of body weight) DMI contents of forage were observed in the intercropping of S10G50+BF and sorghum monoculture (S15) without inoculation, respectively. The average DMI content in different intercropping patterns was enhanced by 22.9 in comparison with sorghum monoculture. Additionally, the DMI content of forage was enhanced by 3.6% after inoculation with BF (Table 2).

### 2.9. Digestible Dry Matter (DDM)

Among different treatments, inoculation of BF in the intercropping pattern of S10G50 produced the highest DDM content of forage (668.01 g kg^−1^ DM). The average DDM content in different intercropping patterns was enhanced by 7.8% in comparison with sorghum monoculture. Also, application of BF enhanced the DDM content of forage by 2.3% (Table 2).

### 2.10. Total Digestible Nutrients (TDN)

The maximum (680.42 g kg^−1^ DM) and minimum (528.82 g kg^−1^ DM) TDN contents of forage were observed in the intercropping of S10G50+BF and sorghum monoculture (S15) without inoculation, respectively. The average TDN content in different intercropping patterns was enhanced by 12.3% in comparison with sorghum monoculture (Table 2).

### 2.11. Relative Feed Value (RFV)

The maximum (142.98%) and minimum (89.17%) RFV values of forage were observed in the intercropping of S10G50+BF and sorghum monoculture (S15) without inoculation, respectively. The average RFV content in different intercropping patterns was enhanced by 33% in comparison with sorghum monoculture. Additionally, application of BF increased the RFV content by 5.9% (Table 2).

### 2.12. Net Energy for Lactation (NEL)

The maximum (1.625 Mcal kg^−1^) NEL content of forage was observed in S10G50+BF. Additionally, the minimum NEL content of forage was observed in the S15, S10 and S15+BF treatments. The average NEL content in different intercropping patterns was enhanced by 12.5% in comparison with sorghum monoculture. Additionally, the NEL content of forage was enhanced by 3.5% after inoculation with BF (Table 2).

### 2.13. Land Equivalent Ratio (LER)

The partial LER of each plant and total LER (LERt) results are shown in Table 3. Based on the obtained results, the highest partial LER of sorghum (0.953) was observed in the intercropping of S15G40 treated with BF. Additionally, the highest partial LER of soybean (0.593) was obtained in the intercropping of S10G40 treated with BF. In all intercropping patterns, the total LER value was greater than one. The highest total LER was recorded in the intercropping of S15G40+BF followed by S10G50+BF (Table 3).

### 2.14. Aggressivity (A) and Competitive Ratio (CR)

In all intercropping patterns, sorghum (As) had positive aggressivity, and soybean (Ag) had negative aggressivity. Similarly, the partial CR of sorghum (CRs) was higher than one and higher than that of soybean (CRg). These results indicate that sorghum was the dominant species in these patterns (Table 3).

### 2.15. Monetary Advantage Index (MAI)

**Figure 3:**
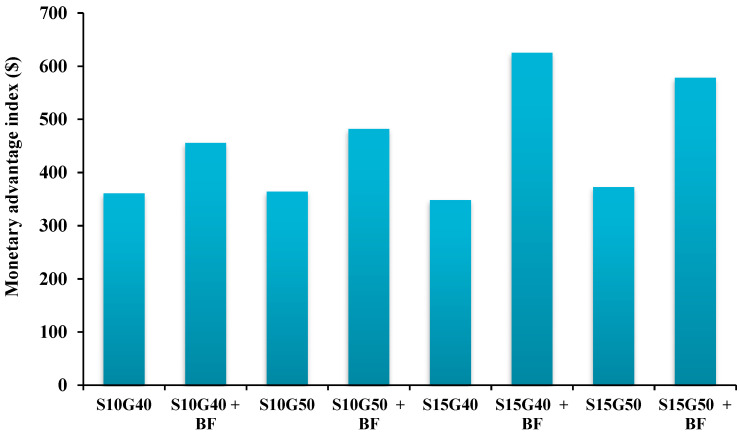
The monetary advantage index (MAI) in different intercropping patterns and bio-fertilizer applications.

Based on the calculated monetary advantage index, all intercropping patterns had positive MAI values. Among different intercropping patterns, the highest (625.37 $) and the lowest (348.24 $) MAI values were measured in the intercropping of S15G40+BF and S15G40 without BF inoculation, respectively. Interestingly, the inoculation of BF in different intercropping patterns enhanced the MAI by 48.1% in comparison with the control (non-application of BF) (Figure 3).

### 2.16. Correlation

**Figure 4:**
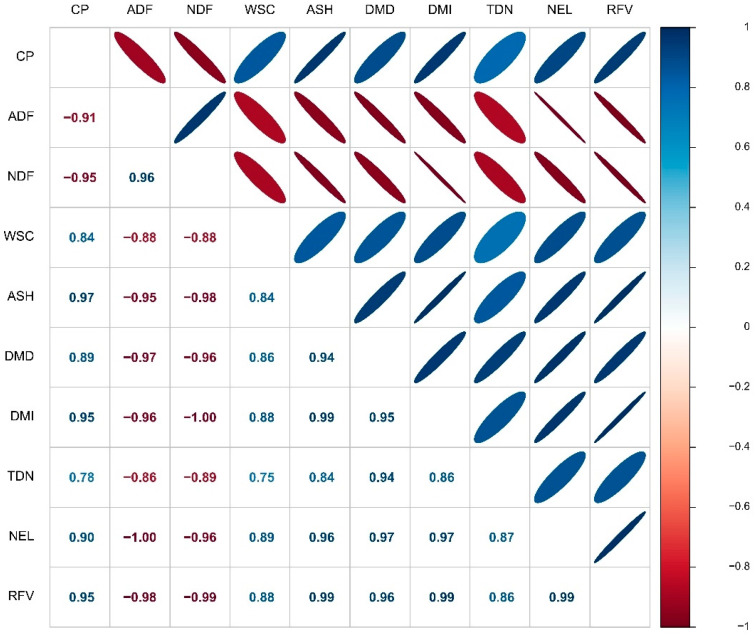
Pearson’s correlation matrix for forage quality characteristics of sorghum. Color ellipses illustrate statistically significant levels. Positive and negative correlations are shown with blue and red, respectively.

Pearson’s correlation results demonstrated that the content of ADF in the sorghum forage had a negative significant correlation with other forage characteristics including CP, WSC, ASH, DDM, DMI, TDN, NEL and RFV (r = −0.88, −0.95, −0.97, −0.96, −0.86, −1.00 and −0.98, respectively). Also, there was a negative significant correlation between NDF and CP, WSC, ASH, DDM, DMI, TDN, NEL and RFV (r = −0.88, −0.98, −0.96, −1.00, −0.89, −0.96 and −0.99, respectively). However, a significant positive correlation was observed between ADF and NDF (r = 0.96) (Figure 4).

## 3. Discussion

The results of the study demonstrated that the forage productivity of sorghum and soybean in monoculture conditions was higher than intercropping patterns. In intercrop patterns, the partial plant density of each crop decreased in the soil area. Therefore, the lower partial forage yield in intercrop patterns could be due to decreasing partial plant density in these cropping patterns in comparison with two plants monoculture. Additionally, in this situation, the higher interspecific competition between companion plants affects the partial forage yield of each plant in comparison with monoculture conditions [20]. Although the partial yield of each plant in different intercrop patterns was lower than both plants’ monocultures, however, it can be concluded that the total forage yield (as shown by land equivalent ratio or LER) in all intercrop patterns was higher than one, representing the advantage of the total productivity of these patterns in comparison with monoculture conditions. The highest total LER value (1.532) was recorded in the intercropping of S15G40 following application of BF, indicating that 53% more area would be required in monoculture to achieve an equal yield of intercropping system. The increasing LER index in intercropping patterns could be explained by increasing complementary processes, thereby improving the environmental use efficiency of resources such as nutrients, water, land, atmospheric CO_2_ and photosynthetically active radiation. In addition, the application of BF enhanced forage productivity due to the role of the BF in enhancing nutrient accessibility and also indirectly exudation of phytohormones (such as indole acetic acid, cytokinin, gibberellic acid) lead to increased photosynthesis rate and plant productivity [21]. Similarly, Javanmard et al. [17] reported that the partial forage yield of maize in different intercrop patterns with legume species (grasspea, berseem clover, bitter vetch and hairy vetch) was lower than maize monoculture; however, the total forage yield was higher than in all intercrop patterns. Similarly, Behrouzi et al. [22] noted that the agronomic traits (plant height, leaf greenness, fresh yield) and dry yield of forage maize were enhanced by BF application.

In all intercropping patterns, sorghum had the higher aggressivity (A) and partial CR index. This means that sorghum has higher capability to optimize the use of available resources compared with soybean, leading to a dominant position [23]. In addition, the higher height of sorghum plants compared to soybeans increased shading on the soybean seedlings, which led to a decrease in received radiation and photosynthesis rate and productivity in this plant.

The monetary advantages index (MAI) represents the economic advantage values among different intercropping systems and also compared with plant monoculture conditions. The results demonstrated that the MAI values in all intercropping patterns were positive, indicating an economic advantage over crop monocultures. The highest MAI values were observed in the intercrop of S15G40 with BF inoculation due to higher LER or total productivity in comparison with sorghum monoculture and other intercrop patterns. Therefore, it can be concluded that although the partial sorghum yield is higher in monoculture conditions, the total yield of two crops and consequently the obtained income from intercropping was higher than sorghum monoculture, which represents the advantage of intercropping patterns in comparison with monoculture conditions.

The qualitative characteristics of forages have a major impact on their value, animal performance and producer profits [17]. The results showed that the CP content in intercrop patterns, especially in S10G50 fertilized with BF, increased in comparison with sorghum monoculture. The CP content in forage crops depends on the available nitrogen. Therefore, the enhancing CP content could be attributed to the increasing nutrient concentration, especially N, as a result of higher activity of N-fixing bacteria and also biological nitrogen fixation by legume plant (soybean) in intercrop patterns and transferring to component plants. Similarly, Stoltz and Nadeau [24] reported that the CP content of maize intercropped with faba bean was higher than maize monoculture, which led to increasing the CP yield by 23% in comparison with plant monoculture.

The concentration of ADF and NDF is considered to be one of the important forage qualities and expresses the amount of cell wall of fodder. The decreasing contents of ADF and NDF enhance the quality of forage. By increasing the ADF and NDF contents, digestible energy levels of forage decrease, and animals are able to consume less forage [25]. Our results showed that the ADF and NDF concentrations were reduced in intercrop patterns, along with BF application. The decreasing ADF and NDF contents could be attributed to improving plant growth characteristics as a result of increasing nutrient availability by application of BF and also enhancing environmental use efficiency in intercropping patterns in comparison with monoculture. These conditions enhanced plant growth and decreased the ADF and NDF contents through reducing the amount of lignocellulosic compounds. Behrouzi et al. [22] reported that the application of bio-fertilizer improved the forage quality of silage maize by reducing the content of ADF and NDF. Javanmard et al. [17] reported that the intercropping of maize with different legume species decreased the content of ADF and NDF and then improved forage quality of maize.

Digestible dry matter (DDM) of forages improves the conversion efficiency of nutrients by livestock and is considered the most important indicator for increasing livestock weight and milk production [17,19]. The results of the study demonstrated that the content of DDM, DMI and TDN was enhanced in intercrop of S10G50 with BF inoculation. Since there is a negative correlation between DMI, TDN and DDM with ADF and NDF (Figure 4), therefore, the increasing of forage digestibility could be explained by decreasing the ADF and NDF content under intercrop patterns following application of BF. Similarly, Sadeghpour et al. [19] noted that the TDN and DMI content in intercropping patterns of barley with annual medic (*Medicago scutellata* L.) was enhanced by decreasing the ADF and NDF content.

Forage ash represents the amount of minerals in plant tissues. The higher ASH content in forages provide more minerals to the livestock, and accordingly, the quality of the fodder will increase. The results showed that the forage ASH content was the highest in the intercrop of S10G50 with BF inoculation, which was due to the higher nutrient accessibility of nutrients in this treatment in comparison with plant monoculture with no-fertilization conditions.

The content of WSC not only plays an important role in increasing the quality of forage but also has a significant effect on resistance to cold and grazing. Soluble carbohydrates constitute a major part of non-structural carbohydrates, which is one of the most important components that determine the quality of fodder, of which the task is to provide energy for rumen microorganisms and maintain the digestive health of animals. The results demonstrated that the highest content of WSC was obtained in the intercrop of S10G50 with BF inoculation. It seems that the higher nutrient concentration in this treatment enhanced the photosynthesis rate, which led to increasing the soluble carbohydrate content in plant cells. Mehrvarz and Chaichi [26] noted that the integrative application of biofertilizer (P-solubilizing bacteria) with chemical fertilizer enhanced the WSC content of forage barley through increasing P uptake and photosynthesis rate. 

RFV, as one of the other forage quality indices, is calculated from DDM and DMI [17]. The RFV value was enhanced in the intercrop of S10G50 with BF inoculation due to higher DDM and DMI as a result of reduction ADF and NDF content in this treatment. Similar results were obtained in NEL values. The increasing NEL in the intercrop of S10G50 with BF inoculation could be explained by decreasing the ADF content in the forage of sorghum. 

## 4. Materials and Methods

### 4.1. Study Site

The research was conducted as a factorial experiment based on a randomized complete block design (RCBD) with three replications at the agricultural research station of Maragheh University, Maragheh, Iran, which is located at 46°16′ E and 37°23′ N, at an altitude of 1485 m sea level, in the 2020–2021 growing seasons. The region has a cold and semi-arid climate. Before the experiment, five soil samples (depth of 0–30 cm) were collected to determine the physical and chemical properties (Table 4). The climatic data of the experimental area are shown in Table 5.

### 4.2. Treatments and Experimental Design

The first factor was different cropping patterns including soybean (*Glycine max* L.) monocultures with densities of 40 and 50 plants m^−2^, sorghum (*Sorghum bicolor* L. Var. speedfeed) monocultures with densities of 10 and 15 plants m^−2^ and intercropping of two plants with the mentioned densities. The second factor was non-application (control) and application of bio-fertilizers. Each plot contained 6 rows with a 4 m length. The type of intercropping of two plants was additive. In this way, soybean was planted on one side of the stack, and sorghum was planted on the other side, with the desired densities. The BFs contained N-fixing bacteria including *Azotobacter vinelandii* strain O4, P-solubilizing bacteria including *Pantoea agglomerans* strain P5 and *Pseudomonas putida* strain P13 and K-solubilizing bacteria including *P. koreensis* strain S14 and *P. vancouverensis* strain S19, which were purchased from Zist Fanavar Sabz Company. The seeds of the two plants were mixed with BF (containing 10^8^ alive and active bacteria) with water in shadow conditions one hour before cultivation. After that, the seeds were sown after drying in shadow conditions. The seeds of the two plants were planted manually and simultaneously in the first week of June. The first irrigation was performed immediately after sowing, and the subsequent irrigations were performed at intervals of 7–10 days by a drip irrigation system.

### 4.3. Measurements

After one week of sorghum flowering, the harvesting was performed after eliminating the marginal effects from a 3.2 m^2^ area. After harvesting, a subsample of 2.0 kg was taken and dried at 65 °C for three days, and then the total dry matter of forage was calculated.

The Kjeldahl method was used for measuring the crude protein (CP) content of the dried forages [27]. For measuring the water-soluble carbohydrate (WSC) content of forage samples, firstly, 0.2 g of each sample were boiled in 40 mL ddH_2_O for 40 min, and fractions were filtered. After that, the samples were transferred to volumetric flasks (50 mL) and brought to 50 mL by the addition of ddH_2_O. The total amounts of WSC were determined as fructose equivalents using the Anthrone colorimetric assay by spectrophotometer at 620 nm [28]. The acid detergent fiber (ADF) and neutral detergent fiber (NDF) concentrations were measured using the procedure described by Van Soest et al. [29]. Other indices, including digestible dry matter (DDM), dry matter intake (DMI), total digestible nutrients (TDN), net energy for lactation (NEL) and relative feed value (RFV) were calculated using the following equations [17]:

DMI = 120/% NDF dry matter basis(1)

DDM = 88.9 − (0.779 × % ADF; dry matter basis)(2)

TDN = (−1.291 × ADF) + 101.35(3)

NE_1_ = [1.044 − (0.0119 × % ADF)] × 2.205 (4)

RFV = % DDM × % DMI × 0.775(5)

The land equivalent ratio (LER) is defined as the land equivalent needed for growing either crop in intercropping systems compared with the land area needed for each crop’s monocultures. The LER values were calculated as follows [30]:

(6) $$\begin{array}{c} \mathrm{LER}={\mathrm{LER}}_{s}{+\mathrm{LER}}_{g}\\ {\mathrm{LER}}_{s}=\frac{Y_{\mathrm{si}}}{Y_{\mathrm{sm}}}\\ {\mathrm{LER}}_{g}=\frac{Y_{\mathrm{gi}}}{Y_{\mathrm{gm}}} \end{array}$$

where LER_s_ and LER_g_ are the land equivalent ratios of sorghum and soybean, respectively; Y_sm_ and Y_gm_ are the yields of sorghum and soybean monocultures; and Y_si_ and Y_gi_ are the yields of sorghum and soybean in intercropping patterns. When the LER value was higher than one, intercropping was more beneficial if compared with monoculture. In contrast, when the LER was lower than one, intercropping had a negative effect on the growth and yield of plants grown in mixtures [31,32].

The aggressivity (A) index was used to evaluate the competitive relationship between two crops in intercropping systems as suggested by Willey [33] using the following formula:

(7) $$\begin{array}{c} A_{s}=\left(\frac{Y_{\mathrm{si}}}{Y_{\mathrm{sm}}\times Z_{\mathrm{si}}}\right)-\left(\frac{Y_{\mathrm{gi}}}{Y_{\mathrm{gm}}\times Z_{\mathrm{gi}}}\right)\\ A_{g}=\left(\frac{Y_{\mathrm{gi}}}{Y_{\mathrm{gm}}\times Z_{\mathrm{gi}}}\right)-\left(\frac{Y_{\mathrm{si}}}{Y_{\mathrm{sm}}\times Z_{\mathrm{si}}}\right) \end{array}$$

where Z_si_ is the sown proportion of sorghum in intercropping with soybean, and Z_gi_ is the sown proportion of soybean in intercropping with sorghum.

Crowding ratio (CR) is another index to evaluate competitive ability between intercropping components. CR gives stronger competitive ability to the species and is more advantageous than other indices. The CR index was calculated by the following equation [31]:

(8) $$\begin{array}{c} CR_{s}=\left(\frac{{\mathrm{LER}}_{s}}{{\mathrm{LER}}_{g}}\right)\times \left(\frac{Z_{\mathrm{gi}}}{Z_{\mathrm{si}}}\right)\\ CR_{g}=\left(\frac{{\mathrm{LER}}_{g}}{{\mathrm{LER}}_{s}}\right)\times \left(\frac{Z_{\mathrm{si}}}{Z_{\mathrm{gi}}}\right) \end{array}$$

where CR_s_ is the crowding ratio of sorghum, and CR_g_ is the crowding ratio of soybean.

None of the above indices provide any information about economic advantage or disadvantage in intercropping systems compared with monoculture. For this reason, an economic index known as the monetary advantages index (MAI) was calculated by applying the following formula [34]:

(9) $$\mathrm{MAI}=\left[\left(Y_{\mathrm{si}}\times P_{s}\right)+\left(Y_{\mathrm{gi}}\times P_{g}\right)\right]\times \left[\frac{LER-1}{LER}\right]$$

where P_g_ is the commercial value of forage sorghum (the current price is 65 $ ton^−1^), and P_s_ is the commercial value of forage soybean (the current price is 91 $ ton^−1^) in Iran.

### 4.4. Statistical Analysis

The normality and homoscedasticity of data regarding the morphological and physiological traits were performed using the Kolmogorov–Smirnov and Levene tests, respectively. All data obtained were subjected to combined analysis of variance (ANOVA) and the significant difference test followed by the LSD test at *p* < 0.05 significance level by SAS version 9.1 (SAS Institute Inc., Cary, NC, USA). In addition, for drawing the Pearson correlation matrix (correlation plot) between main forage quality characteristics including CP, ADF, NDF, ASH, WSC, DDM, DMI, TDN, RFV and NEL contents, R software v3.2.4 was used.

## 5. Conclusions

The results of the study demonstrated that the total forage productivity (calculated by LER) in all intercropping patterns following application of bio-fertilizers was higher than sorghum and soybean monoculture. The higher LER values in all intercropping patterns enhanced the monetary advantage index of these cropping patterns in comparison with plant monoculture. Additionally, the forage quality characteristics such as CP, ash, WSC, DMI, DDM, TDN, RFV and NEL improved in intercrops, especially in the S10G50, and inoculation with bio-fertilizers. Generally, the intercropping of S10G50 with bio-fertilizer inoculation are highly recommended for acceptable productivity, higher quality and economic revenue of forage compared with sorghum monoculture.

## Figures and Tables

**Table 1:** The quality characteristics of forage in different cropping patterns and bio-fertilizer application.

Treatments		CP (g kg^−1^ DM)	ADF (g kg^−1^ DM)	NDF (g kg^−1^ DM)	WSC (g kg^−1^ DM)	ASH (g kg^−1^ DM)
Control	S10	83.5 f	365.33 ab	595.6 b	181.73g	48.13 f
	S15	90.8 f	377.43 a	622.56 a	180 g	51.06 f
	S10G40	124.7 cd	305.9 de	499.73 c	228.37 cd	90.86 cd
	S10G50	137.3 b	277.4 g	464.9 ef	221.57 de	104.3 ab
	S15G40	115.03 d	318.08 cd	510.53 c	217.47 def	77.33 e
	S15G50	133.3 bc	311.1 de	491.4 cd	219.83 def	93.30 cd
Bio-fertilizer	S10	102.4 e	333.73 c	578.70 b	214.80 ef	54.16 f
	S15	103.3 e	356.43 b	578.63 b	208.23 f	53.46 f
	S10G40	123.6 cd	297.23 ef	478.86 de	245.20 ab	95.46 cd
	S10G50	149.6 a	258 h	449.36 f	251.16 a	113.2 a
	S15G40	133.03 bc	301.63 def	498.26 cd	238.60 bc	86.33 de
	S15G50	137.2 b	287.86 fg	479.16 de	236.8 bc	97.3 bc

Different letters in each column indicate significant differences at the 5% level according to LSD test.

**Table 2:** The quality characteristics of forage in different cropping patterns and bio-fertilizer application.

Treatments		DDM (g kg^−1^ DM)	DMI (g kg^−1^ of Body Weight)	TDN (g kg^−1^ DM)	NEL (Mcal kg^−1^)	RFV (%)
Control	S10	611.36 g	20.21 ef	553.38 g	1.367 g	95.64 ef
	S15	596.54 h	19.30 f	528.82 h	1.317 g	89.17 f
	S10G40	660.70 de	24.57 cd	618.58 de	1.499 cde	121.33 d
	S10G50	672.9 b	25.60 b	655.37 b	1.574 ab	135.47 b
	S15G40	641.21 ef	23.63 d	602.85 ef	1.467 ef	117.39 d
	S15G50	646.65 de	24.57 cd	611.87 de	1.486 de	122.78 cd
Bio-fertilizer	S10	629.52 f	20.78 e	582.65 f	1.426 f	101.21 e
	S15	604.4 gh	20.85 e	541.85 gh	1.343 g	97.52 e
	S10G40	657.45 cd	25.24 bc	629.77 cd	1.548 bc	128.59 bc
	S10G50	668.01 a	26.83 a	680.42 a	1.625 a	142.98 a
	S15G40	654.02 cde	24.10 cd	624.09 cde	1.511 cde	122.14 cd
	S15G50	664.75 bc	25.08 bc	614.86 bc	1.547 bc	129.16 bc

Different letters in each column indicate significant differences at the 5% level according to LSD test.

**Table 3:** The land equivalent ratio (LER), aggressivity (A), and competitive ratio (CR) in different intercropping patterns and bio-fertilizer application.

Treatments		LERs	LERg	LERt	As	Ag	CRs	CRg
Control	S10G40	0.901	0.548	1.449	0.353	−0.353	1.644	0.608
	S10G50	0.908	0.539	1.447	0.368	−0.368	1.683	0.594
	S15G40	0.906	0.405	1.311	0.502	−0.502	2.240	0.447
	S15G50	0.812	0.529	1.341	0.283	−0.283	1.534	0.652
Bio-fertilizer	S10G40	0.921	0.593	1.514	0.328	−0.328	1.552	0.644
	S10G50	0.938	0.592	1.530	0.392	−0.392	1.588	0.630
	S15G40	0.953	0.579	1.532	0.374	−0.374	1.646	0.608
	S15G50	0.934	0.546	1.481	0.354	−0.354	1.710	0.585

Different letters in each column indicate significant differences at the 5% level according to LSD test.

**Table 4:** Physico-chemical properties of field soil (average of two years).

Soil Texture	Sand (%)	Silt (%)	Clay (%)	OM (g kg^−1^)	EC (ds m^−1^)	pH	CEC (Cmolc kg^−1^)	N (g kg^−1^)	P (mg kg^−1^)	K (mg kg^−1^)
Sandy clay loam	56.3	16.3	27.4	8.1	1.17	7.73	26.6	0.84	9.43	553.17

**Table 5:** Monthly average temperature and total monthly precipitation in 2020 and 2021 growing seasons and long-term averages in the experimental area.

Year	April	May	June	July	August	September
Monthly average temperature (°C)						
2020	11.8	19.1	24.2	28.0	25.1	23.8
2021	16.3	21.3	27.2	28.3	28.1	23.02
2-year mean	14.1	20.2	25.7	28.1	26.6	23.4
10-year mean	12.9	18.5	24.4	28.1	27.5	22.7
Total monthly precipitation (mm)						
2020	63.3	12.0	2.6	0.1	1.2	0.0
2021	12.01	13.3	0.01	3.10	0.02	0.1
2-year mean	37.6	12.7	1.3	1.6	0.6	0.05
10-year mean	41.8	19.9	1.5	0.7	0.3	1.8

## Data Availability

The datasets generated and analyzed during the current study are available from the corresponding author upon reasonable request.

## Abbreviations

Abbreviation Lists	Full Form
A	Aggressivity
ADF	Acid detergent fiber
BF	Bio-fertilizer
CP	Crude protein
CR	Competitive ratio
DM	Dry matter
DDM	Digestible dry matter
DMI	Dry matter intake
LER	Land equivalent ratio
MAI	Monetary advantage index
NDF	Neutral detergent fiber
NEL	Net energy for lactation
RCBD	Randomized complete blocks design
RFV	Relative feed value
TDN	Total digestible nutrients
WSC	Water soluble carbohydrate
